# NK Cells in the Pathogenesis of Chronic Obstructive Pulmonary Disease

**DOI:** 10.3389/fimmu.2021.666045

**Published:** 2021-05-04

**Authors:** Yafei Rao, Yanqing Le, Jing Xiong, Yuqiang Pei, Yongchang Sun

**Affiliations:** Department of Respiratory and Critical Care Medicine, Peking University Third Hospital, Beijing, China

**Keywords:** natural killer cells, chronic obstructive pulmonary disease, COPD, acute exacerbations, cigarette smoke, pathogenesis

## Abstract

Chronic obstructive pulmonary disease (COPD) is a prevalent chronic airway disease with varied frequencies of acute exacerbations, which are the main cause of morbidity and mortality of the disease. It is, therefore, urgent to develop novel therapies for COPD and its exacerbations, which rely heavily on understanding of the pathogenesis and investigation for potential targets. Current evidence indicates that natural killer (NK) cells play important roles in the pathological processes of COPD. Although novel data are revealing the significance of NK cells in maintaining immune system homeostasis and their involvement in pathogenesis of COPD, the specific mechanisms are largely unknown. Specific and in-depth studies elucidating the underlying mechanisms are therefore needed. In this review, we provided a brief overview of the biology of NK cells, from its development to receptors and functions, and outlined their subsets in peripheral blood and lungs. Then we reviewed published findings highlighting the important roles played by NK cells in COPD and its exacerbations, with a view of providing the current state of knowledge in this area to facilitate related in-depth research.

## Introduction

Chronic obstructive pulmonary disease (COPD) is a prevalent chronic airway disease with increasing morbidity and mortality globally (1–3). The global incidence of COPD was estimated to achieve 3.9%, and caused a mortality of 41.9 per 100 000 individuals in 2017 (2). A recent cross-sectional study in mainland China revealed an overall prevalence of 8.6% of spirometry-defined COPD in adult populations (≥20 years old) (3). COPD is a preventable and treatable disease featured by persistent airway inflammation and parenchymal destruction, mostly caused by cigarette smoking. Clinically, COPD is usually manifested as exertional dyspnea, cough and/or sputum production (4, 5), and acute exacerbation of these symptoms, i.e., AECOPD, represents the main cause of COPD-related disabilities and mortality and accelerates disease progression (6, 7). Currently, main therapeutic interventions for COPD include non-pharmacological management (smoking cessation, influenza and pneumococcal vaccinations, rehabilitation), and pharmacological therapies with bronchodilators and/or inhaled corticosteroids. However, none of the available medications has been shown to modify disease progression and reduce mortality (5). It is, therefore, urgent to develop novel therapies for COPD and its exacerbations, which rely heavily on understanding of the pathogenesis and identification of potential targets.

Studies have shown that immune-infiltrating cells play a pivotal role in the airway inflammation and lung destruction of COPD, including neutrophils, macrophages, lymphocyte subsets, and dendritic cells (8, 9). NK cells, as innate immune cells, are considered to be the first line of defense mechanism for the human body against infections and tumors (10). More recent evidence has implicated NK cells in maintaining immune homeostasis and in pathogenesis of COPD (11, 12). However, the specific mechanisms are still elusive (13, 14). Specific and in-depth studies elucidating the underlying mechanisms are therefore needed. In this article, we provided an overview of the biology of NK cells, their subsets in peripheral blood and the lungs, and current knowledge on the potentially important roles played by NK cells in COPD and its exacerbations.

## Human NK Cell Development

Over the past 40 years, great achievements have been made in the field of research on NK cells. Studies in humans revealed that the bone marrow (BM) and secondary lymphoid tissues (SLTs) such as tonsils, spleen, and lymph nodes (LNs) are essential organs for the differentiation and development of NK cells (15, 16). Different stages of this process were determined by unique surface biomarkers (15). During the initial phase, hematopoietic stem cells (HSCs) give rise to CD34^+^CD133^+^CD38^-^CD90^-^CD45RA^+^ lymphoid-primed multipotential progenitor (LMPP) (17), and then, LMPP progress into common lymphoid progenitors (CLPs) with the expression of CD38, CD7, CD10, and CD127 (18–21). Hereafter, CD122 marks the irreversible transition of CLPs into NK lineage, and then develop into its mature form *via* CD56 expression (22). Some works have also categorized the development of NK cells into six stages according to the studies pertaining to BM and LNs development (15, 16). CD3ϵ^−^CD7^+^CD127^+^cell subgroup defines the transformation of NK cells from stage 1 through stage 2a. Expression of IL-1R marks the initiation of stage 2b. Then NK cells come to stage 3 with the expression of activating receptors involving NK cell group 2D(NKG2D), CD335 and CD337. During stage 4, NK cell development is comprised of two sections: stage 4a and stage 4b. NK cells in Stage 4a are NKP80^-^ that have high expression of NKG2D, CD335, CD337, inhibitory NKG2A and CD161. Besides, this stage also exhibits high expression of CD56 (CD56^bright^). On the other hand, NK cells in stage 4b are NKP80^+^ while sustain CD56^bright^ status (23, 24). Stage 5 of development is denoted by a proportion of NK cells with downregulated CD56 (CD56^dim^) and upregulated CD16 (25). Finally, stage 6 of the development, also known as terminal maturation, occurs following expression of CD57 (26).

## Subgroups of Human NK Cells in Peripheral Blood and Lungs

NK cells are defined as CD3^-^CD56^+^ cells, which make up approximately 5–15% of the circulating lymphocytes. NK cells are subdivided into two main subpopulations, CD56^bright^ CD16^-^ and CD56^dim^ CD16^+^, based on expression of the surface marker CD56 and CD16. CD56^bright^ CD16^-^ NK cells, accounting for about 10% of peripheral blood (PB) NK population, mainly produce cytokines, including interferon-gamma (IFN-γ), interleukin (IL)-10, tumor necrosis factor-α (TNF-α), granulocyte-macrophage colony-stimulating factor. CD56^dim^ CD16^+^ NK cells are the predominant (90%) peripheral blood (PB) NK population, which are highly cytotoxic by producing a large amount of perforin and granzymes, expressing Ig-like receptors (KIRs), and inducing antibody-dependent cytotoxicity (ADCC) effects, but with limited ability to produce cytokines (27–29).

**Figure 1:**
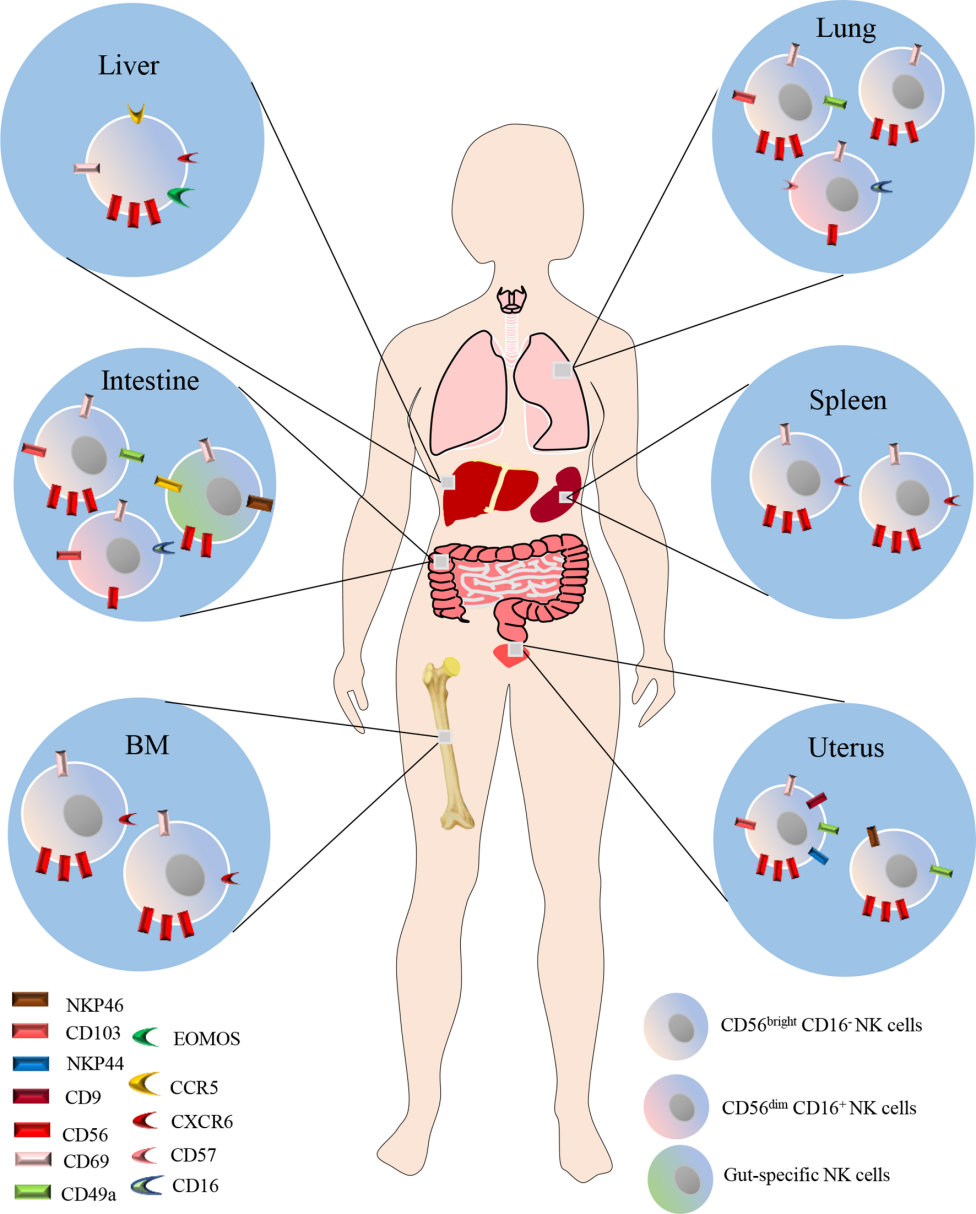
NK cells also reside in peripheral tissues including liver, lung, spleen, BM, intestine and uterus under steady-state conditions. The majority of NK cells in the lung, BM, LNs and spleen are CD56^dim^ CD16^+^, while CD56^bright^ CD16^-^ NK cells predominate in the liver, gut and uterus. CD56 ^bright^CD69^+^ CXCR6^+^ NK cells define the BM and spleen resident NK cells. While CD56 ^dim^CXCR6^+^CD69^+^NKP46^+^ NK cells define LN resident NK cells. In humans, NK cells constitute approximately 5-20% of the CD45-positive lung lymphocytes, and are located in lung parenchyma. Most lung NK cells show a mature CD56^dim^CD16^+^ phenotype which takes a proportion of 80%, whereas CD56^bright^CD16^−^ and CD56^dim^CD16^−^ cells make up the remaining 20%, in equal ratios. A minority of the lung NK cells are tissue-resident, and could be specifically defined by CD49a^+^CD69^+^CD103^+^ cells, which constitute 20% of lung NK cells.

In recent years, emerging evidence indicates that NK cells also reside in peripheral tissues including liver, lung, spleen, BM, intestine and uterus under steady-state conditions (**Figure 1**). Studies indicated that the majority of NK cells in the lung, BM, LNs and spleen are CD56^dim^ CD16^+^, while CD56^bright^ CD16^-^ NK cells predominate in the liver, gut and uterus (30–34). NK cells in the spleen, BM and LNs comprise 5-20%,4% and 2-5% of the total lymphocyte population, respectively. CD56 ^bright^CD69^+^ CXCR6^+^ NK cells define the BM and spleen resident NK cells, which make up a proportion of 9-51% in BM NK cells, and 28-69% in spleen NK cells, respectively. While CD56^dim^CXCR6^+^CD69^+^NKP46^+^ NK cells define LN resident NK cells, accounting for 43-67% of the LN NK cells (34, 35). Dogra et al. found that the frequencies of NK cells in BM, spleen and lung were higher than those in LNs and gut, and interestingly, they also demonstrated that tissue sites shaped the functions of NK cells, especially the potential for cytokine production (31). In humans, NK cells constitute approximately 5-20% of the CD45-positive lung lymphocytes, and are located in lung parenchyma (28). Most lung NK cells show a mature CD56^dim^CD16^+^ phenotype which takes a proportion of 80% (29), whereas CD56^bright^CD16^−^ and CD56^dim^CD16^−^ cells make up the remaining 20%, in equal ratios (12, 36). However, it should be noted that the majority of lung NK cells are circulating cells, though hypofunctional, otherwise are similar to PB NK cells. A minority of the lung NK cells are tissue-resident, and could be specifically defined by CD49a^+^CD69^+^CD103^+^ cells, which constitute 20% of lung NK cells (34). Due to the difficulty of obtaining human lung NK cell samples, the research in human lung NK cells is very limited. Thus, further investigations are needed to fill the gap in understanding of human lung NK cells in pulmonary diseases.

## Human NK Cell Receptors and Their Functions

**Figure 2:**
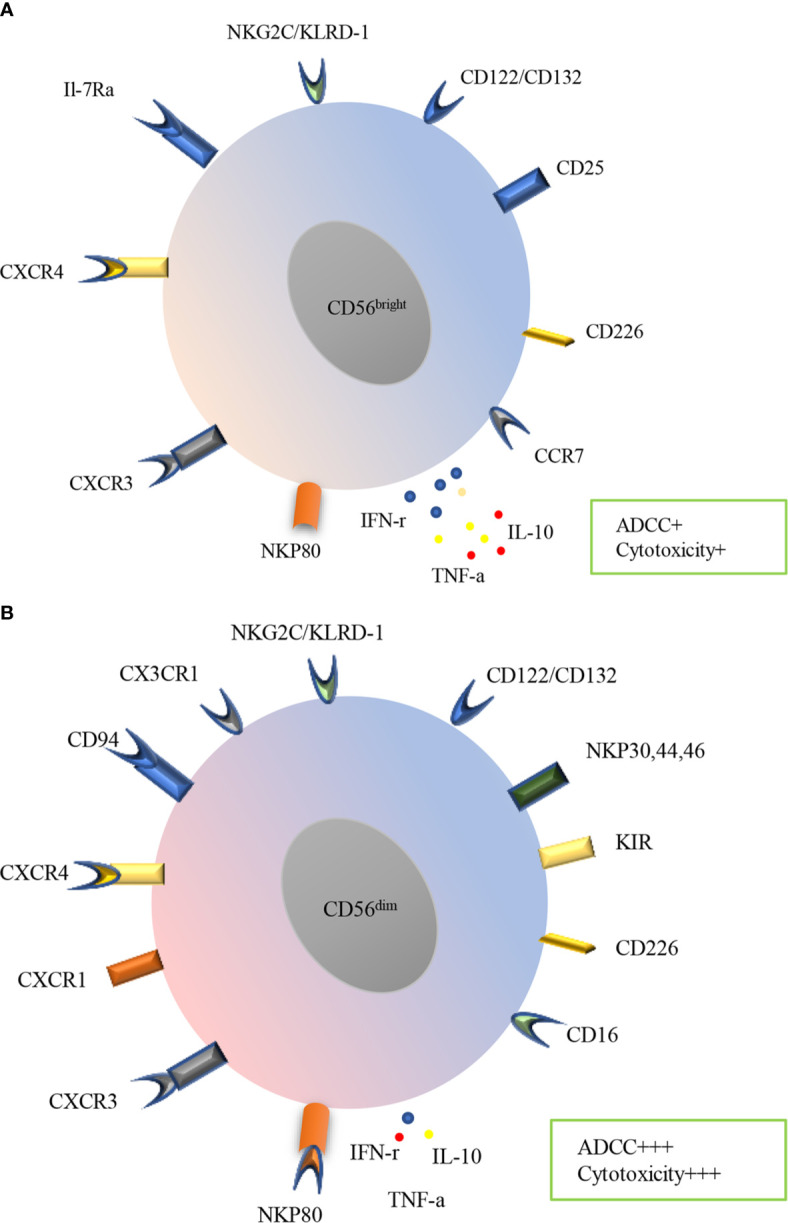
Surface receptors and functions of PB human NK cells subtypes. Human NK cells are divided into CD56^bright^ **(A)** and CD56^dim^ **(B)** subsets in PB, which express a variety of receptors, including inhibitory, activating, cytokine, chemokine receptors and death receptors. CD56^bright^ subset mainly expresses CCR7, CD226, CD25, CD122/132, NKG2C/KLRD-1, IL-7Ra CXCR4, CXCR3and NKP80, meanwhile, CD56^dim^ subset mainly expresses CD16, CD226, KIR, NKP30, NKP44, NKP46, CD122/CD132, NKG2C/KLRD-1, CX3CR1 CD94, CXCR4, CXCR1, CXCR3. CD56^bright^ subset produces more IFN-γ, TNF-α and IL-10 while less ADCC and cytotoxicity. In contrast, CD56^dim^ subset produces less cytokines, however prominent ADCC and cytotoxicity.

Human NK cells express a variety of receptors, including inhibitory, activating, cytokine, chemokine receptors and death receptors (**Figure 2**). Inhibitory receptors are expressed when immune surveillance is normal, among them are killer KIRs such as KIR2DL1, KIR2DL2/3, KIR3DL1, KIR3DL2, KIR2DL5 (expressed by subsets of CD56^dim^ NK cells) and KIR2DL4 (expressed by all mature CD56^dim^ NK cells), CD94: NKG2A heterodimers and immunoglobulin-like transcript 2(ILT-2). Recently, several other inhibitory receptors have also been reported, including programmed cell death protein 1 (PD-1), T cell immunoglobulin and immunoreceptor tyrosine-based inhibitory motif domain (TIGIT), cytotoxic T lymphocyte antigen-4 (CTLA-4), lymphocyte-activation gene 3 (LAG-3), killer cell lectin-like receptor subfamily G member 1 (KLRG1), CD161, T cell immunoglobulin domain and mucin domain-3 (TIM-3) (37). All mature NK cells may also express the inhibitory receptor IRp60 and p75/AIRMI. Inhibitory receptors mainly recognize classical and non-classical major histocompatibility complex (MHC) class I molecules (38, 39). Regulation of the balance between inhibitory and activating receptors determines NK cell effects. NK cells are activated by tumor or infected cells lacking inhibitory receptor ligands or having abundant activating receptor ligands. NK cells will undergo activation if the activating signals surpasses the MHC class I inhibitory receptors (40–42). Activating receptors include natural cytotoxicity receptors (NKp30, NKp44, and NKp46), natural killer group 2C (NKG2C), NKG2D, CD16, KIR2DS1, KIR2DS2/3, KIR2DL4, KIR3DS1, KIR2DS5, KIR2DS4, NKRP-1, CD226 as well as coreceptors (2B4, NTB-A, and NKp80) (10, 38, 39). NK cells may also express many other cytokine and chemokine receptors, including IL-2Ra, IL-2Rb/IL-2Rc, c-Kit, IL-7Ra, CXCR1, CXCR3, CXCR4, CCR4, CCR7, IL-18R, ChemR23 and CX3CR1. In addition, death receptors such as TNF-related apoptosis-inducing ligand (TRAIL), Fas and Apo1 as well as ligands including Fas ligand and CD40L have also been reported in NK cells (39). Knowledge of these receptors and their functions is essential for understanding the role of NK cells in diseases including COPD.

## NK Cells in Viral and Bacterial Lung Infections

NK cells play a vital role in lung immune response to respiratory viral and bacterial infections. During the early phase of influenza infection in mice, NK cells accumulate in the lung to clear the virus *via* IFN-γ production, adaptive immune cell activation, ADCC and direct lysis (43, 44). Studies have demonstrated that patients with genetic deficiency with loss of NK cell function suffered from recurrent viral infection (45–47). However, NK cells may not always protective. Deletion of NK cells promote the survival of mice infected with high-dose influenza viruses *via* mitigating lung immunopathology, suggesting the destructive role of NK cells in influenza infection. It seems likely that NK cells play a dual role in influenza infection.

Recent evidence indicates that NK cells may play an increasingly vital role in bacterial infections. During infection with *Mycobacterium tuberculosis*, NK cells upregulated CD69, IFN-γ and perforin expression for promotion of host defense against the bacteria. However, it is still unclear whether NK cells employ cytotoxic lysis to restrict *Mycobacterium tuberculosis*, and therefore further investigations are needed (48–51). In another study, lung NK cells protect the host against *K. pneumoniae* infection *via* IL-22 and IFN-γ production. Similarly, lung NK cells defend against *Pseudomonas aeruginosa* infection *via* NKG2D expression and IFN-γ production (52, 53). Yoshihara et al. found that NK cells facilitate host defense against *Staphylococcus aureus* infection through IFN-γ and TNF production (54, 55). Similarly, lung NK cells could also protect against *Haemophilus influenzae* through IFN-γ production. Overall, current knowledge shows that NK cells play a beneficial role in the restriction of bacterial infection (56).

## NK Cells in Animal Models of COPD

### Frequency of NK Cells in COPD

An earlier study by Motz et al. found no significant change of lung NK cells after 6-month cigarette smoke (CS) exposure in a mouse model of COPD (57). Similar results were confirmed by Wortham et al. who observed no difference in the number of lung NK cells between 6-month CS-exposed and air-exposed mice (58). Additionally, another study reported that short-term CS exposure (4 days) did not increase the frequency of NK cells in the lung (59). On the contrary, Stolberg et al. found that lung NK cells showed elevated frequency after 4-day CS exposure (60). The reasons for conflicting results from these studies are not clear, but may be explained by differences in experimental protocols, CS exposure times and doses.

### Activating Receptors of NK Cells in COPD

The study by Wortham et al. found that the activating receptors of NK1.1, NKG2D, and CD244 in lung and spleen NK cells did not differ between 6-month CS-exposed and air-exposed mice (61). In contrast, Stolberg et al. found that after a short-term of 4-day CS exposure, lung NK cells showed elevated CD69 expression, which could be mitigated by CCR4 deletion. Additionally, they found that short-term CS exposure could significantly induce the expression of retinoic acid early transcript 1 protein (RAET1) (NKG2D ligand) in lung airway epithelium (60). Other studies also confirmed that long-term CS exposure could significantly induce the expression of NKG2D ligand expression including RAET1 and Mult1, which were shown to contribute to pulmonary emphysema (58, 62). Similarly, in a spontaneous COPD mouse model, Finch et al. observed increased percentage of CD69^+^ lung NK cells (14). These discrepancies in activating receptors of NK cells in COPD models maybe explained by differences in CS exposure times and doses, the receptors studies, and the compartments of NK cells. Collectively, these data indicate that activating receptors of NK cells are altered and may play an important role in lung inflammation and emphysema of COPD.

### Inhibitory Receptors of NK Cells in COPD

Thus far, there has been limited data on the inhibitory receptors of NK cells in COPD models. In the study by Wortham et al., the inhibitory receptor CD94 as heterodimerized with NKG2A/C subunits in the lung and spleen showed no difference between 6-month CS-exposed and air-exposed mice (58). Indeed, further experimental evidence is needed to elucidate the potential role of NK cell inhibitory receptors in the pathological mechanisms of COPD.

### Altered Effector Functions of NK Cells in COPD

The study by Wortham and his colleagues found that NK cells from 6-month CS-exposed mice showed elevated cytotoxicity toward NKG2D ligand RAET1ϵ (58). Similarly, Motz et al. found that lung and spleen NK cells from 8-week CS-exposed mice demonstrated elevated cytotoxicity following Ly49D or NK1.1 challenge. In this study, they also found that lung and spleen NK cells in CS-exposed mice showed higher IFN-γproduction following IFN-γ-inducing cytokine challenge including IL-12 or IL-18, or both. Particularly, they found that IFN-γ^+^ NK cells decreased significantly after IL-12 treatment following smoking cessation (57). In another study, Finch et al. observed that lung NK cells from 8-week CS exposed mice showed elevated cytotoxicity toward lung epithelial cells in relative to control group. In this article, they also found that DCs were necessary for the priming of NK cells to acquire cytotoxicity potential following CS exposure, which could be abrogated by blocking IL-15 (14). Additionally, another study demonstrated that CS could prime NK cells for the production of IL-17A (59). Stolberg et al. found that lung NK cells from mice exposed to CS for 4 days showed elevated IFN-γand CXCL10 production, which could be abrogated by CCR4 deletion. Also, they found that CCR4 was required for NK cells’ contacting with lung CD11c^+^and CD11^+^MHCII^+^ cells (60). In summary, these studies imply that the CS exposure may exaggerate lung epithelial cell injury through increased NK cell cytotoxicity and cytokine production.

### NK Cells in AECOPD of Mice

Other studies demonstrated that NK cells produced much more IFN-γ in CS-induced COPD models after viral infection or after viral pathogen-associated molecular patterns (PAMPs) challenge (63). Additionally, IFN-γ^+^lung NK cells frequency from in CS exposed mice for 8 weeks responded differently to different bacterial PAMPs: increase for LPS, no difference for HKLM and ST-FLA, decrease for pam3CSK4 (57). Another study found that infection of mice with virus in six-month CS exposure showed exaggerated inflammation and airway epithelial damage. However, this augmented damage could be mitigated in NKG2D deletion mice, indicating the pivotal role of NKG2D in viral infection induced COPD exacerbations (58). In contrast, one study by Mian et al. showed that CS inhibited IL-15 mediated NK cells activation, manifested as decreased CD69, NKG2D and Granzyme B expression as well as NK cell cytotoxicity following poly I:C stimulation (64). In line with this, Pichavant et al. found that the NK cells in 12-week CS-exposed mice showed defective immune response upon *S. pneumoniae* challenge (65). In all, the study of NK cells in AECOPD of mice is still in its infancy, there is an urgent need to assess the role of NK cells in AECOPD with more innovative approaches.

## NK Cells in Human COPD

Evidence indicates that immune-infiltrating cells play a pivotal role in the pathogenesis of COPD (8, 9). NK cells act as a proinflammatory population during the immune response (12). Theoretically, NK cells could cause the injury to the lung, and thus COPD pathogenesis. However, their precise role in the pathogenesis of COPD remained elusive with discrepant studies in humans (**Table 1**). Alterations of NK cells, as manifested by surface marker including inhibitory receptors, activating receptors (**Table 2**), as well as effector functions occur in stable COPD patients and smokers.

**Table 1:** Studies of human NK cells in COPD.

References	Patients	Compartment	Functional assay	Observations in NK cells	Key findings
Wang et al. (66)	HNS 21 Smokers 21 CuS-COPD 14 ExS-COPD 10	PB	Yes	Decreased CD158e1 expression in smokers and CuS-COPD compared with HNS	Hyperfunction of NK cells in COPD
Chen et al. (67)	HNS 16 COPD 40	PB	Yes	Increased NK cells in COPD, decreased FPR3 expression	Immune imbalance in COPD
Pascual et al. (68)	HNS 13 COPD 66	PB	No	Increased NK cells in severe-to-very severe COPD VS mild-moderate COPD	Immune imbalance in COPD
Hodge et al. (13)	HNS 25 Smokers 16 CuS-COPD 30 ExS -COPD 41	PB	Yes	Increased CD94 and Granzyme B expression in COPD compared with HNS; no difference of CD69 between COPD and HNS group	Hyperfunction of NK cells in COPD
Fang et al. (69)	HNS 12 COPD 19	PB	Yes	Elevated NK cells in COPD patients, decreased IFN-γ production	Hypofunction of NK cells in COPD
Finch et al. (14)	ExS 4 CuS 15 CuS-COPD 14 ExS -COPD 16	Lung	Yes	Increased NK cells cytotoxicity in COPD compared with HNS	Hyperfunction of NK cells in COPD
Wang et al. (66)	HNS 5 Smokers 10 CuS-COPD 5 ExS -COPD 6	Induced sputum	Yes	Increased CD69^+^ and/or CD25^+^ NK cells in CuS-COPD and ExS -COPD patients compared with HNS	Hyperfunction of NK cells in COPD
Hodge et al. (13)	HNS 19 Smokers 12 COPD 33	BALF	Yes	Increased NK cells frequency, cytotoxicity, Granzyme B, CD94 in COPD patients compared with HNS and smokers	Hyperfunction of NK cells in COPD
Prieto et al. (70)	HNS 50 COPD 60	PB	Yes	Decreased cytotoxicity in COPD patients	Hypofunction of NK cells in COPD
Hughes et al. (71)	HNS 32 Smokers 14	PB	Yes	Decreased cytotoxicity in heavy smokers, but normal ADCC	Hypofunction of NK cells in COPD
Phillips et al. (72)	HNS 22 Light/moderate smokers 12 Heavy smokers 12	PB	Yes	Declined cytotoxicity in heavy smokers, normal in mild smokers	Hypofunction of NK cells in COPD
Freeman et al. (73)	Smokers 6 Mild COPD 14 Severe COPD 15	lung	Yes	Elevated cytotoxicity in COPD	Hyperfunction of NK cells in COPD
Urbanowicz et al. (16)	HNS 5 Smokers 10 COPD 11	Induced sputum	Yes	Elevated frequency and higher perforin and granzyme B production in CD56^bright^CD16^-^ subset of COPD patients compared with HNS and smokers.	Hyperfunction of NK cells in COPD

**Table 2:** Activating and inhibitory receptors in COPD of human.

Receptor type	Name	Compartment	Findings	Reference
Activating receptors	CD69 and or CD25	PB/Induced sputum	Increased CD69^+^ and/or CD25^+^ in smokers CuS-COPD and ExS-COPD compared with HNS	(66)
	NKG2C	PB	Elevated frequency NKG2C^+^ NK cells in frequently exacerbation COPD compared with occasional exacerbated COPD	(70)
	NKG2C/NKG2A	PB	No difference of NKG2C^+^ NK cells between COPD and HNS, decreased NKG2A^+^ in COPD compared with HNS	(68)
	CD69	PB	No changes of CD69^+^NK cells between COPD and HNS	(13)
Inhibitory receptors	CD158e1	PB	Decreased CD158e1^+^NK cells in smokers and CuS-COPD compared with HNS	(66)
	CD94	PB	Decreased CD94^+^ NK cells in CuS-COPD compared with HNS	(13)
	CD94	BALF	No difference between smokers and HNS, decrease in CuS-COPD compared with HNS	(13)
	CD158a/CD158b	PB	Decreased CD158a^+^ and CD158b^+^ in COPD compared with HNS	(69)

### Frequency of NK Cells

As the disease progresses, the frequency of NK cells may also change. One study by Richard et al. indicated that the percentage of induced sputum NK cells increased significantly in COPD patients compared with healthy non-smokers (HNS) and smokers. CD56^bright^CD16^-^ induced sputum NK cells subset also increased significantly in COPD patients in relative to the two other groups (74). Another study observed an elevated percentage of PB NK cells in COPD patients compared with HNS (69). Similarly, Chen et al. also observed an elevated percentage of PB NK cells in cigarette smoking-related COPD patients (67). In addition, another study by Olloquequi et al. showed increased NK cells in lung lymphoid follicles (75). However, NK cells may not always increase. Several studies gave opposite results showing decreased frequencies of PB CD16^+^ NK cells in smokers as compared to nonsmokers, which persisted years after ceasing smoking (76, 77). Similarly, one study demonstrated that the percentage of NK cells decreased significantly in COPD patients compared with HNS. The CD56^dim^CD16^+^ PB NK cells subset also reduced in COPD patients in relative to smokers and HNS with a corresponding elevation of the CD56^bright^CD16^-^ cells (78). Interestingly, Pascual et al. pointed out that PB NK cell frequency showed no difference between HNS and COPD patients disregarding disease severity (68). Hodge et al. confirmed that there were no changes in the percentage of PB NK cells between HNS, smokers, current smoker COPD and ex-smoker COPD. However, this study observed an elevated frequency of bronchoalveolar lavage fluid (BALF) NK cells in COPD patients compared with HNS (13). Taken together, currently available data indicate that the NK cell population increases in the pulmonary compartment of COPD, but varies in the blood circulation.

### Activating Receptors of NK Cells

There are a variety of activating receptors on the surface of NK cells, which are crucial for the function of NK cells (39). Wang et al. observed elevated frequency of activated (CD69^+^CD25^-^; CD69^+^CD25^+^; CD69^-^CD25^+^) NK cells in PB of healthy smokers, current smokers and ex-smokers with COPD in relative to healthy non-smokers, and this was positively correlated with the number of cigarettes smoked. Additionally, they found increased proportion of activated induced sputum NK cells in current smokers with COPD and ex-smokers with COPD compared with healthy non-smokers. Interestingly, the proportion of activated NK cells was elevated in current smokers with COPD compared healthy smokers. These data implied that activating receptors in NK cells were close related with COPD state and cigarettes smoked. However, no differences were observed in NKG2D expression between groups (66). More recently, it was demonstrated that there were no differences in the percentage of NKG2C^+^ PB NK cells between COPD patients and HNS, and no correlation was observed with disease severity, smoking status. However, they found decreased NKG2A^+^ NK cells in COPD patients in relative to HNS. Yet, NKG2A^+^ PB NK cells level were in no correlation with disease severity, smoking status and exacerbations frequency (68). Additionally, Hodge et al. and his colleagues reported that there were no changes in the percentage of CD69^+^ PB NK cells between COPD patients and HNS groups (13). Overall, these studies gave absolute different observation, thus a profiling of NK cells receptors are urgently needed.

### Inhibitory Receptors of NK Cells

Studies have found a variety of crucial inhibitory receptors in the surface of NK cells which are crucial for NK cells function (39) One study by Wang et al. demonstrated that the proportion of PB NK cells expressing CD158e1 was significantly decreased in healthy smokers and current smokers with COPD in relative to HNS (66). In line with this, Hodge et al. found that both PB and BALF NK cells’ inhibitory receptor CD94 was also significantly decreased in current smoker COPD patients compared with HNS. However, there was no alteration of PB NK cells CD94 in healthy smokers compared with HNS. Meanwhile, no difference was observed in BALF NK cells regarding CD94 expression between ex-smoker COPD and HNS (13). In contrast, Tang et al. found increased inhibitory receptor CD158a^+^and CD158b^+^ PB NK cells in COPD patients, which were negatively correlated with pulmonary function, indicating that NK cell inhibitory receptors may contribute to COPD progression (69). There is still no consensus on reliable observation about inhibitory receptors of NK cells concerning COPD. which may due to different locations of NK cells, receptor types, smoking status, disease severity. Thus, in-depth research is urgently need.

### Altered Effector Functions of NK Cells

NK cells may perform effector functions through producing perforin, granzyme indirectly and killing target cells through cytotoxicity directly (12). Urbanowicz et al. found that the CD56^bright^CD16^-^ NK cells subpopulation in induced sputum produced significantly more perforin and granzyme B in COPD patients compared with HNS and smokers. However, they observed no difference in the percentages of CD56^bright^CD16^-^ and CD56^dim^CD16^+^ NK cells producing only perforin and no granzyme B (74). One study by Hodge et al., who reported that there were a higher percentage of PB and BALF NK cell expressing granzyme B in COPD patients compared with HNS (13). Similarly, there were significantly higher percentage of PB CD56^dim^CD16^+^subset expressing only perforin and no granzyme B in COPD patients compared with the two other group (78). In contrast, Tang et al. found decreased IFN-γ production in PB NK cells of COPD patients compared with HNS (69) Additionally, the percentage of PB NK cells expressing both perforin and granzyme B decreased obviously in COPD patients in relative to smokers and HNS, while not the CD56^bright^CD16^-^ subset. However, no difference between the percentage of PB CD56^bright^CD16^-^ or CD56^dim^CD16^+^subsets expressing only granzyme B and no perforin was observed. Similarly, the proportion of CD56^bright^CD16^-^ PB NK expressing only perforin and no granzyme B showed no differences between the three groups (78). Additionally, no changes were observed regarding Granzyme A and perforin expression in PB NK cells between COPD patients and HNS (13). Even though these results involving cytokines production are of great promising, they are preliminary and definitely need further investigation.

In addition to cytokine production, it is worth mentioning that cytotoxicity, another mechanism through which NK cells perform direct killing effect, play a vital role in COPD pathogenesis. In one article, Urbanowicz et al. found that the cytotoxicity of PB NK cells from COPD patients was lower than those from smokers and HNS, and similarly decreased cytotoxicity was observed in smokers compared with HNS. Particularly, they observed a positive correlation between the cytotoxicity of PB NK cells and the lung function (78). The decreased cytotoxicity of PB NK cells in COPD patients was also confirmed by Prieto and his colleagues, and this decline could be rescued by Glycophosphopeptical treatment (70). In line with this, Hughes et al. found that heavy smokers showed decreased PB NK cell cytotoxicity, but normal ADCC (71). Similarly, Phillips et al. also reported that PB NK cells from heavy smokers displayed declined cytotoxicity, which was normal in mild smokers (72). However, several studies observed contrary findings. One study by Hodge et al., found that BALF NK cells showed elevated cytotoxicity in COPD patients compared with HNS (13). Similarly, a higher cytotoxicity of lung NK cells was also observed in COPD patients compared with smokers, and these NK cells induced severer injury of the lung epithelium. This article indicated that increased cytotoxicity in COPD was driven by lung NKs, not lung epithelial cells. The study also demonstrated that dendritic cells (DCs) from COPD patients could significantly enhance NK cell cytotoxicity through IL-15Rαtrans-presentation, suggesting that blocking IL-15 may protect lung epithelial cells from injury (14). Another study reported a positive correlation between enhanced stress-induced NK cell cytotoxicity and COPD severity, suggesting the contribution of heightened lung NK cell cytotoxicity in emphysema progression (73). All these observations implies that the intervention of cytotoxicity, cytokine release may be potential targets of COPD treatment.

### Adhesion Molecules of NK Cells

Richard et al. found that CXCR3 and very late antigen-4(VLA-4) expression in CD56^bright^CD16^-^ and CD56^dim^CD16^+^ NK cells elevated significantly in COPD patients compared with smokers (74). Another study by Chen et al., observed that intracellular expression of formyl peptide receptor (FPR3) in PB NK cells was markedly decreased in COPD patients compared with HNS. Particularly, this FPR3 expression showed a positive correlation with pre-bronchodilator FEV1/FVC ratio and predicted FEV1 percentage. FPR3 expression in COPD patients showed obvious elevation after 1-year treatment, suggesting the promising role of NK cell FPR3 in COPD therapy (67). Thus, searching for potential responsible molecule may be promising in the therapy of COPD.

### NK Cells in AECOPD of Human

Exacerbations are vital events in the management of COPD for their negative effect on health status, hospitalization and readmission rates as well as disease progression, which are primarily triggered by viral and/or bacterial infections (5). In one study, higher levels of PB NKG2C^+^ NK cells were closely associated with the number of exacerbations, implying a potential role in predicting COPD exacerbations (68). Another study reported that NK cells from COPD patients were not responsive to S. *pneumoniae*, suggesting function defect of NK cells in COPD patients (65). Up till now, the role of NK cells in AECOPD remains under-appreciated, and in-depth investigation holds promise for better understanding the immune mechanisms underlying infection-related acute exacerbation and deterioration of the disease.

### Concluding Remarks

As innate immune cells, NK cells are considered to be the first line of defense mechanism for the human body against infections and tumor. Available data demonstrate that NK cells probably play a pivotal role in COPD and its exacerbations. Though advances have been made in revealing the potential involvement of NK cells in COPD, there still remain discrepancy involving NK cells frequency, activating and inhibitory receptors, effector functions, which may be attributable to different compartment of NK cells, different stages of disease in human and different dose of CS exposure in mouse model of COPD. Thus, further studies are urgently needed to elucidate the mechanisms of NK cells in the pathogenesis, endotypes and acute exacerbations of COPD. Particularly, the investigators should pay more attention to cross talks between adaptive immune cell and NK cells, NK cells and epithelial cells, which may shed light on the mechanisms of lung damage (emphysema), a hallmark of COPD. Also, investigators should put emphasis on researches involving inhibitory, activating receptors and chemokine receptor in NK cells and its ligand in epithelial cells, thus develop more potent agonists/antagonists, which may pave the way to facilitate the translation of such a promising strategy into clinical use for therapy of COPD and AECOPD.

## Author Contributions

YR analyzed the data and prepared the manuscript. YS contributed to the scientific expertise and manuscript preparation. YL, JX, and YP participated in the preparation of the manuscript. All authors contributed to the article and approved the submitted version.

## Funding

This work was supported by the National Natural Science Foundation of China (81770040, 81970041) and Natural Science Foundation of Beijing Municipality (7192224).

## Conflict of Interest

The authors declare that the research was conducted in the absence of any commercial or financial relationships that could be construed as a potential conflict of interest.
